# Scoping the Need for a Tailored mHealth App to Improve Health and Well-being Behavioral Transformation in the Police: Exploring the Views of UK Police Workers via Web-Based Surveys and Client Meetings

**DOI:** 10.2196/28075

**Published:** 2021-08-31

**Authors:** Emma Swanston, Andy Pulman, Huseyin Dogan, Jane Murphy, Fiona Bitters

**Affiliations:** 1 Faculty of Science and Technology Bournemouth University Poole United Kingdom; 2 Faculty of Health and Social Sciences Bournemouth University Bournemouth United Kingdom; 3 Hampshire Constabulary Southampton United Kingdom

**Keywords:** nutrition, food, behavior change, mobile health, police, lifestyle management, well-being, mobile phone

## Abstract

**Background:**

Police officers often work long, unsocial hours in a highly pressurized environment and may experience difficulties in managing their health and well-being. Their jobs can be highly stressful and feature unusual working hours and multiple shift patterns. When considering the policing environment of 2021, many roles that were previously the domain of warranted officers are now being carried out by nonwarranted police staff equivalents. These police staff roles are relatively new to policing but put staff under some of the same stresses as police officers. A UK police force requested help to investigate technologies that could be used to improve health and well-being and research how these technologies could be used to measure and track health behavior change.

**Objective:**

Historical research studies need to be appraised in light of this new policing environment, and new research also needs to include this shift in dynamics when considering aspects of policing, including their health and well-being. This study explores police officer and staff attitudes toward and their use of existing health-related technology, highlights existing practices, and gathers views about how technology could be used more effectively.

**Methods:**

A web-based survey was completed by police officers and staff (N=213) during the initial period of the UK lockdown in 2020. The survey was designed to find the solutions that participants used outside of those supplied by their employer, identify issues or problems, and find what they would like a hypothetical app to focus on. Additional requirements data were captured through client meetings, including discussions concerning previously attempted solutions and those currently in place. Thematic analysis was undertaken to identify the key themes.

**Results:**

Attitudes toward and uses of existing health-related technology were captured, and existing practices were highlighted. Participants identified a need for an app to consider that a user was on shift—an important point, as many issues and problems with elements of their health and well-being involved shift work. Data also highlighted that a multifunctional tool would be more beneficial to participants than focusing on just 1 element. The key features and four domains were identified for app coverage. The prioritized order of importance of the four domains was activity, food and diet, sleep, and fluid intake.

**Conclusions:**

For police officers and staff, research data suggest that there is a previously unidentified requirement for a mobile app that could provide an easily accessible platform for them to use, regardless of the current location; one that could provide guidelines on diet, lifestyle habits, and health behavior to help the user make informed decisions to assist in personalized behavior change. Notably, one which is multifunctional and which also aligns effectively with the irregular shift patterns of its users.

## Introduction

### Policing and Health and Well-being

Police officers often work long, unsocial hours in a highly pressurized environment, and as a result, they can experience difficulties in managing their health and well-being [1,2]. Their jobs can be highly stressful and may feature unusual working hours and irregularly changing shift patterns [3]. Police officers are more likely to experience stress because of exposure to dangerous situations and traumatic events [4] and other stress-related issues, such as working in understaffed environments [5]. Stress can have multiple knock-on effects, including insomnia, fatigue, and lapsed concentration—all of which make performing the job harder than it already is, thereby adding to the original problem [6]. When considering the policing environment of 2021, many roles previously the domain of warranted officers are now carried out by nonwarranted police staff equivalents. These roles are relatively new to policing but put staff under some of the same stresses as police officers. However, stress is not the only mental health condition that police officers and staff are more likely to experience. A 2012 study found that 46.7% of US police officers had required help for mental health conditions during their lifetime [7]. In the United Kingdom, a 2018 survey of serving police officers and staff suggested that symptoms of posttraumatic stress disorder (PTSD) and complex PTSD (CPTSD) might be present in as many as 1 in 5 workers [8]. A companion study noted that CPTSD was more common than PTSD in police officers, and the data supported a cumulative burden model of CPTSD [9].

Police officers also have a greater risk of being overweight or obese and experiencing long-term chronic conditions, such as cardiovascular disease [10] and cancer [11]. Risk factors are heightened by working in a high-stress environment [4], which increases the likelihood or severity of these issues [12,13]. A 1996 study of police officers found that prolonged exposure to body armor and mobile patrols could increase the risk of back pain [14]. Comparing later data from a December 2020 internal commissioning client well-being report confirms this as still being an issue—with muscular skeletal reasons being the fourth highest cause of sickness absence. This is an issue that could have a major effect over a longer period, as low back pain is a health problem leading to the greatest disability in the United Kingdom [15].

Although some risk factors relating to health and an individual’s risk of certain health issues are uncontrollable (age, gender, and genetic family history of disease), others are modifiable risks and can be managed by individuals to reduce risk. These include managing diet, alcohol intake, and the amount of physical activity undertaken [16] to help improve health and well-being.

### Policing and Health and Well-being (During a Pandemic)

In January 2020, the World Health Organization Emergency Committee declared a global health emergency based on the growing case notification rates of COVID-19 [17]. Since the start of 2020, a number of different lockdown laws have been enacted globally and within the United Kingdom—fluctuating periods of restrictions, lockdowns, and rule relaxations made law [18]. Global and UK policies for policing during COVID-19 have similarly altered during this time [19]. Emergent research during the last year suggests that stress levels of policing during COVID-19 will further exacerbate health and well-being issues [20,21]. COVID-19 policing was hypothesized by Stogner et al [20] as a significant stressor for officers and compounding the general and organizational stresses associated with the occupation. A web-based survey of 2567 police officers across 5 European countries showed that law enforcement agencies and police officers were unprepared for the potential mental stress of policing during COVID-19 [21]. The research paper by De Camargo [22], using data from 18 UK interviews during summer 2020, suggested that unsupportive line managers and colleagues—demonstrated by examples of ridicule and the downplaying of the virus’s seriousness—could only elevate officers’ stress and anxiety, warranted or not.

### Rationale and Aims

During 2020, a UK police force approached our team and requested help in investigating new technologies that could be used to help them keep track and manage various aspects of health. The initial research aims were as follows:

1. Exploring current police officer and staff attitudes toward and their use of existing health-related technology.
2. Highlighting existing practices and gathering views about how technology can be used more effectively in this area.

### Theoretical and Practical Contributions

This paper discusses the initial scoping phase of our research, which took place during the first lockdown period in the United Kingdom in 2020. It describes the methods used to undertake these aims, collecting data from 213 participants from the UK police force via web-based surveys and from face-to-face client meetings. We then discuss the results in three thematic sections—exploring attitudes toward and their use of existing health-related technology, highlighting existing practices, and gathering views about how technology could be used more effectively in this area. The results are discussed in light of the current research.

As already noted, both previous studies and future research in health and well-being for this user group need to be considered in the context of a policing environment where many roles previously the domain of warranted officers are now carried out by nonwarranted police staff equivalents. The policing environment of 2021 is not the same as it was earlier. Our new and unique contribution to the research pool is conducting initial scoping in the midst of the pandemic and being mindful of the needs to include both this impact and the shift in work dynamics and roles when considering aspects of health and well-being for all police workers and the unique demands of their jobs.

## Methods

### Study Design

Methods chosen were considered with the participants in mind—those that could be completed in 1 session, where time could be set aside when they were free. We used a human-centered approach to optimize understanding and accommodate the perspectives of potential users [23].

### Participants, Recruitment, and Consent

Data were gathered in the southern region of the United Kingdom where the commissioning organization was based. Participants were recruited through a purposive sampling strategy that targeted personnel within the organization. Survey participants were recruited via a gatekeeper from the commissioning organization. The gatekeeper role was to initiate communication between the researcher and police officers and staff who wished to participate—via distribution of study literature—without compromising anonymity or affecting the veracity of web-based responses. Participants were voluntary and remained anonymous to both the gatekeeper and the organization.

Although other methods were initially considered to capture the original requirements, because of complications arising from COVID-19 in terms of logistics, lockdown restrictions, and operational pressures on the commissioning force, requirements were also captured from client meetings.

### Procedures and Measures

A web-based survey was chosen as the method of data collection. This was owing to the initial UK COVID-19 lockdown period restricting in-person meetings, the number of people we were able to gather data on, and the shortened time to complete surveying (in comparison with undertaking interviews). The participants provided consent before the survey was completed. Using a web-based survey as the main method of data collection allowed initial ideas and requirements to be documented in depth and helped tailor prototype ideas to accurately reflect participant needs. Content validity (also referred to as face validity) indicates whether a questionnaire appears logical to a group of experts [24]. A pilot version was sent to 5 participants working in the field, who completed the questionnaire and provided feedback during an interview. To ensure suitable content validity, questions were asked around their ability to understand questions, whether categories allowed users to give the answer they wanted, and general ease of understanding. This feedback was used to make changes and improvements before it was sent back to the client for additional feedback. Improvements were then implemented into a revised version, which was sent to the client for further feedback. All participants completed the survey in less than 30 minutes. This was important as it was required to not take up too much participant time because of operational availability. Once the final version was confirmed, a second test run was completed by the same participants (n=5) who had taken the draft survey, but this time on Google Forms.

The survey included a *mixture* of open-ended, qualitative and closed questions. Qualitative questions were used to collect information relating to the features and functions that participants used most on apps and their benefits and limitations, also reflecting on issues and problems which a new app could potentially help them with. Closed questions were used to collect demographic statistics and details on currently used technology, such as accessible devices, operating systems used, and device preferences. Owing to the nature of the questions being asked, such as providing opinions on health and well-being apps and listing popular and unpopular features, we did not use a Likert scale within the closed questions included.

Client meetings were intended as a combination of discussion on clients’ expectations, their background with the project or similar projects, and answering preprepared questions. The initial meeting took place with a client representative and a representative from Bournemouth University and covered the background to the project and what direction it might be expected to go in. Subsequent meetings took place with a researcher and the client representative and covered potential existing solutions, solutions the client organization had already tried or had in place, data gathering, and expectations for evaluating developing prototypes.

### Sample Size

The recommended sample size was calculated to ensure that the number of participants for the survey was acceptable. The sample size was calculated with a 95% confidence level and a margin of error of 5%. As the intended audience for the final product was a UK police force, the workforce value supplied by the client organization was rounded to the nearest 1000 and used to calculate an ideal sample size. The value used for the workforce was 5000. Owing to the high workforce number, it was expected that a confidence level of 95% and margin of error of 5% might not be possible because of restrictions with accessing participants. A margin of error of 5% was calculated to be used as the goal value to reach (95% confidence level+5% margin of error; n=375 participants), but a margin of error of 7.5% was accepted as the lowest value (95% confidence level+7.5% margin of error; n=165 participants) acceptable for this study [25].

### Data Analysis

Survey data were analyzed and summarized using descriptive statistics. Data were tested for normal distribution and then presented as means, SDs, ranges, and percentages. For age, as under 21 years and over 60 years did not have confirmed lower and upper values, respectively, 18 years and 65 years were used to calculate the mean, SD, and range (18 years being the lowest age of UK employment and 65 years being the current age of UK retirement).

Open questions were thematically analyzed using a deductive approach that focused on the areas covered in the questions (such as design, functionality, and content). A generic qualitative approach to thematic analysis was used [26] with interresearcher interpretation. Following familiarization with the qualitative data, a member of the team charted the initial themes. A second researcher subsequently familiarized themselves with the data and initial themes. They developed a coding scheme using an analytical framework that combined a priori issues from the original surveys and emerging themes [27]. We attended to the principles of sampling, saturation, negative cases, confirmation, and logical progression throughout so that bias and any other errors identified were eradicated during inquiry.

## Results

### Overview

The number of participants was 213, which was above the lower threshold for an acceptable number of participants for the sample size [23]. With this number of participants and an estimated population of 5000, there is a 95% confidence level with a final margin of error of 6.5%.

### Participant Characteristics

We recruited 37.1% (79/213) male and 62.9% (134/213) female participants. The “other” or “prefer not to say” option was also included within the gender question—no responses were received. The mean age of the participants was 41.9 (SD 10.0) years (Table 1).

**Table 1:** Participant demographic data (n=213).

Characteristics				Participants
**Age (years)^a^**				
	Value, mean (SD; range)		41.9 (10.0; 21-65)	
	**Age group, n (%)**			
		Under 21	0 (0)	
		21-30	26 (12.2)	
		31-40	75 (35.2)	
		41-50	67 (31.5)	
		51-60	38 (17.8)	
		Over 60	7 (3.3)	
**Gender** **, n (%)**				
	Male		79 (37.1)	
	Female		134 (62.9)	
	Other or prefer not to say		0 (0)	

^a^As under 21 years and over 60 years did not have confirmed lower and upper values, respectively, 18 years and 65 years were used to calculate the mean, SD, and range (with 18 years being the lowest age of UK employment and 65 years being the current age of UK retirement).

### Attitudes Toward and Use of Existing Health-Related Technology

#### Technology Use

Most (206/213, 96.7%) of the participants noted they had access to a smartphone, compared with 78.4% (167/213) for a PC or laptop and 58.2% (124/213) for a tablet. Alongside these data, 78.9% (168/213) of participants reported that out of the options available to them, they used a smartphone the most. Regarding operating systems, 56.3% (120/213) of participants said their smartphone ran on Android as opposed to 43.2% (92/213) running Apple iOS.

Out of all the participants, 43.2% (92/213) did not use a smart watch or device of any kind; therefore, at this point in time, a large number of potential users would not be able to use or take advantage of features targeted specifically at those devices. Reasons given for not using these ranged from lack of awareness of the options available, the technological skills required to make use of them, and cost restrictions. Where some participants had previously used these devices, there were also reasons given for nonuse currently, such as reliability: “Used to use Fitbit but broke it and haven’t got a new one” [P145]. Issues with interoperability, features, and functionality: “I used to have an Apple Watch but found constant messages coming through to it was distracting when trying to work or relax and therefore no long wear it” [P188]. There were also a range of other explanations:

The electronic wrist device gave me an upset stomach and a small irritated patch of skin. I don’t feel all the tech is good for my body.

P40

#### Apps in Use

Participants noted a number of different mobile health (mHealth) apps that they currently used to help with aspects of their health and well-being (Table 2).

**Table 2:** Mobile health apps mentioned in relation to usage (n=213).

Categories		Participants, n (%)
**General health**		
	Fitbit^a^	21 (9.8)
	Garmin^b^	16 (7.5)
	Apple Health^c^	9 (4.2)
	Samsung Health	4 (1.8)
	Huawei Health	1 (0.4)
	Google Fitness	1 (0.4)
	ProFit^d^	1 (0.4)
	Virgin Health–Max Buzz	1 (0.4)
**Activity**		
	MyFitnessPal	20 (9.3)
	Strava	18 (8.4)
	MapMy (Walk, Run, or Ride)	7 (3.2)
	Runkeeper	2 (0.9)
	Peloton	2 (0.9)
	Fitnotes	1 (0.4)
	Interval Timer	1 (0.4)
	Fitbit Coach	1 (0.4)
	Pacer	1 (0.4)
	Polar Beat	1 (0.4)
**Food or diet**		
	Weight Watchers	2 (0.9)
	NHS^e^ Well Man	1 (0.4)
	NHS BMI Tracker	1 (0.4)
	NHS Calorie Checker	1 (0.4)
	Noom	1 (0.4)
	Nutracheck	1 (0.4)
	Slimming World	1 (0.4)
**Rest, relaxation, or mental health**		
	Headspace	7 (3.2)
	Mindfulness UK	2 (0.9)
	Breathe2Rrelax	1 (0.4)
	Calm	1 (0.4)
	Waking Up	1 (0.4)
**Fluid**		
	Drink Free Days	1 (0.4)
**Other**		
	COVID-19 Symptom Tracker	1 (0.4)
	GP24	1 (0.4)
	Tinnitus Therapy	1 (0.4)

^a^Including the Fitbit app.

^b^Including connect.

^c^Including workouts and activity.

^d^Including VeryFitPro.

^e^NHS: National Health Service.

#### Barriers to Use and Success

Barriers to engagement and adherence highlighted included the risk of organizational infrastructure causing difficulties in improving aspects of health and well-being. These could be physical building limitations:

Not enough different options in the [Police Investigation Centre] PIC canteens.

P112

Our police station has no adequate, clean, hygienic area to prepare food, so the default is to snack or binge on treats. Our police station has no canteen, so no healthy options are available even though the organisation want and stress that officers should eat well and not rely on fast food.

P162

Or also managerial issues affecting the ability to change:

I have suggested that the app includes screen breaks and breaks from work. The problem with this is that whilst senior management seem to support this, day to day management [sgts and inspectors] do not, so if you are seen getting up and taking breaks you are considered not to be working. For example just two days ago I was told by a sgt that I was not entitled to a lunch break even though we are being encouraged to eat lunch away from our screens! The app sounds great, but without support from line managers it’s not going to work.

P188

I do not believe that technological solutions are the biggest factor in solving staff health and wellbeing. A flexible approach within management for the health and wellbeing of staff would be a more beneficial solution.

P129

Data also suggested that there could be an in-built resistance for some who were not in the right mindset to want to engage with a technological solution offered to them:

Pointless applications, how have we, the human race, survived this long without being told that we need to drink water by some artificial device. I know if I haven’t slept enough, I’m tired. These are issues that do not need managing, they are issues invented by the manufacturers of needless devices.

P98

Apps will only make you think about your wellbeing. It’s down to whatever inspires the individual to get them off the sofa and start moving, to give them the availability [time management] to home cook and to afford to eat well/healthy. Recipes are good and prompts are just that, prompts.

P54

In addition, some participants also offered a variety of other reasons, which might have caused them to avoid engaging with a new solution offered on a smartphone. This again highlighted the technology experience concerns:

Money, Privacy, Guilt induction by failure to see progress.

P45

I don’t know how to set it up I’m not very technically minded.

P203

Some participants noted an element of technology fatigue (where they were having to use technology most of the time): “Fed up with everything being online, using my time up to log in/passwords/checking etc” [P63]. Another barrier to success in the area of emotional and mental well-being was the continual demands of the job:

But this is a job like no other [few other occupations including other emergency services get so emotionally, verbally and physically abused] and get micro examined, as the Police. That will take a change in [the small number of] societies attitude and behaviour that would make the greatest help to mental and emotional wellbeing, not to mention time and effort.

P54

### Highlighting Existing Practice—Employer Initiatives

The client organization had investigated other solutions to help improve employee health and well-being. They had participated in the Virgin Pulse Corporate Global Challenge [28], but participation in this had now finished. As this had run for a limited period, the opportunities of longer-term effects from this had in effect stopped at that point in time, as noted by the following responses:

Time limited - health challenge.

P110

Global challenge finished so could no longer log into the application.

P151

In client meetings, another of the existing solutions listed was investing in retreats focusing on mental health and well-being. However, this particular aspect was currently limited to the numbers who could take advantage against the total number of staff working for the force. No mention was made of these events in the survey responses.

### Gathering Views About How Technology Could Be Used More Effectively

#### App Coverage

The areas of health and well-being that participants said they felt they could benefit from having support from the most in an app were prioritized by response. The four highest prioritized domains were food and diet (76/213, 35.6%), activity (68/213, 31.9%), sleep (27/213, 12.6%), and fluid intake (27/213, 12.6%; Textbox 1). Other lower scoring, suggested areas for support included relaxation techniques and rest breaks (2/213, 0.9%), general health and fitness goals (1/213, 0.4%), differing aspects of mental health (1/213, 0.4%), and specific gender-related health issues (1/213, 0.4%). No preference (10/213, 4.6%) accounted for the other responses.

**Domains to support health-related behavior change identified for app coverage.**

**Food and Diet**

- “Shift work making organising meals more difficult. My own lack of self-discipline of making better choices. The convenience and availability of fast food and takeaways.” [P166]
- “Keeping it simple and using realistic meals that are quick and easy. Other apps have very overly complicated meals.” [P145]
- “Stress Eating.” [P120]

**Activity**

- “My own motivation - I used to get my exercise from my previous job as I was on my feet all day, but now I have a sedentary job, I have gained weight as a result, but my diet hasn’t changed an awful lot.” [P59]
- “Time constraints.” [P104]
- “Feeling bad when I don’t exercise!” [P24]

**Fluid Intake**

- “I know that I don’t usually drink as much water as I should and drink a lot of coffee. I also don't track how much water I drink.” [P32]
- “Because we are in an office all day you forget to go to the kitchen to get water.” [P188]
- “Lack of prompting to drink.” [P102]

**Sleep**

- “Medication and insomnia at times.” [P184]
- “Not being able to switch off my mind/relax.” [P117]
- “Shift patterns and concerns about work.” [P182]

#### What Is Missing Currently

Notably, when analyzing responses, participants wanted an app to take into consideration the fact that a user was on shifts—an important point, as many issues and problems participants had within each of the 4 identified domains involved shift work:

I like eating and work shifts, meals aren’t always practical as suggested on apps.

P37

I’m a shift worker and find that none of these apps really know how to advise those who work shifts. They are aimed at those who work mon-fri 9-5.

P61

Web-based data collected also highlighted that a multifunctional tool would be more beneficial to participants than focusing on just one element:

It would be great to have a one stop shop for health and wellbeing.

P46

Having an app that combines all aspects is what is required. There are apps for counting calories, but don’t give enough inspiration for exercise or keeping active daily.

P185

Considering a wider focus for an app, rather than just focusing on one or two specific subsections, was suggested within the data because of the nature of the research problem and the environment and job requirements of participants:

Other apps are all about exercise, but don’t help with the food aspect.

P185

Most apps are aimed at very active very fit people, I am inactive, have arthritis and need an app to set realistic targets that build over time with strength, repetition and confidence. I usually can never achieve the targets which is off putting.

P14

#### Key Features

##### Dashboard and Visualization

Participants wanted the ability to visualize how their behavior might be changing and how aspects they used could be personalized, such as those relating to changes in individual shift patterns:

When you have beaten a personal best you get a notification or you have a dashboard of your best efforts to see easily.

P144

There are lots of people that work shifts so could benefit from helpful advice explaining how to shift sleep pattern during night shifts and what to eat, when to eat, etc.

P61

##### Goal-Setting and Gamification

The option of setting regular goals within each of the four domains identified (eg, fluid intake, snacks avoided or consumed over the course of a day, or planned physical activities achieved) was also described as a useful feature within an app. Nearly half of the participants (106/213, 49.7%) said that they did not enjoy gamification features in an app. Of the 28.6% (61/213) of participants who said they enjoyed gamification features, the most popular types of gamification were goals (44/61, 72%), daily challenges (42/61, 69%), and leaderboards (40/61, 65%) with competition-style gamification, such as teams (23/61, 38%) and experience points (22/61, 36%) being the least popular:

I would like to see goals / medals / points achieved for weight loss and same for good nutrition, which also shows the improvement scale over a period of time.

P108

I really dislike group participation. I am very self-disciplined, and I like to set my own targets and goals and strive to beat my own PB’s. I enjoy exercise, but enforced group participation would demotivate me to a point of non-engagement.

P5

##### Diary Options

Participants thought that the ability to enter diary-based information into the app would be useful, such as notes associated with differing shift times. One barrier to using the existing apps highlighted was their adherence to keeping daily routines the same and not allowing for users who worked nonstandard shift patterns:

Most applications of this nature cater for either athletes or people that work regular hours during the week, they do not cater for people who work weekends, overnight or irregular/shifts. Most of the research is not catered for those who have no choice so they get regular sleep, eat at odd times.

P6

##### Customizable Notifications

Of the 113 participants who used a health or well-being app, 47 (41.6%) said that they checked an app multiple times during a day and that they thought that notifications would help them to remember to do certain tasks. Shift-related customized reminders were also suggested to be helpful:

It couldn’t change to my shift patterns, for instance on nights I don’t need a 07:00hrs reminder to drink water.

P7

## Discussion

### Principal Findings

#### Attitudes Toward and Their Use of Existing Health-Related Technology

This research has highlighted that for participants, a mobile app rather than other mediums would provide the best platform for user access. Smartphone apps are increasingly considered useful health promotion tools owing to their accessibility and scalability [29] and with a view to integrating health within a work-based framework [30]. Penetration of smart devices (including watches) lags behind phones [31], with more associated barriers involved in their wider use at this point—highlighted by participants in comparison with more widely adopted and user-friendly phone technology—although this might change as the market matures.

An increasing number of apps and technologies for managing aspects of health and well-being have been launched over the past 10 years [32]. There are currently a number of health and fitness solutions available on multiple platforms in personal use by police officers and staff, as evidenced by our data (Table 2). The fact that most apps focus on one particular element of health and well-being is notable. They have not been designed to address the specific issues that police officers and staff face, nor are they configurable for the types of routines and working patterns that they regularly encounter [33]. Recent literature reviews, such as a 2020 review of research on gamification and mHealth apps for emergency service personnel and police officers across 6 major databases, have highlighted a lack of literature in this area for these groups [34].

Spanning both police officers and staff, our research has highlighted concerns that the working environment makes it harder for those affected to make healthy choices, such as physical building limitations and managerial challenges. Therefore, the problem includes not only thinking of a solution to help manage personalized risk issues but also ensuring that the solution would not be intrusive for the user during and outside of work, to counteract the in-built resistance some participants noted when engaging with this type of media. As became clear from the data, solving this issue will not be easy because of the fact that one solution would not work effectively for all police officers and staff. Barriers to usage and workplace limitations for those who work in the police provide a challenge to solving this issue. Considering activity, perceived pressure of work and organizational culture appears to be a sturdy barrier to reducing sedentary time [35]. Police officers in previous studies have expressed a need for more opportunities to take breaks and more encouragement from managers or supervisors [36], as some of our participants also noted. In terms of pre- and postpandemic mental health, particularly challenging are the potential difficulties of highlighting and raising this topic within a culture where broaching mental health difficulties have sometimes been viewed as a sign of weakness or undesirable topic to discuss [37].

#### Highlighting Existing Practice

As a part of our project, we performed 2 reviews of research concerning different domains of police officers and staff health and well-being—separate but often interlinked domains thematically identified in our research to date. This also included a review of current mHealth technology in use by police forces to help address these domains. Article length precludes a more detailed discussion, but we are able to summarize some notable points here in relation to our data analysis at this stage.

The client organization had already investigated solutions to help improve employee health and well-being. They had participated in the Virgin Pulse Corporate Global Challenge [28], with each employee being offered a smart tracker. A desktop or mobile app is used to track the progress of teams throughout the challenge and a self-health assessment used initially—to help users understand the goals and improvements they should aim to make [38]. Notably, this was limited to the period of the actual challenge rather than a long-term intervention with the issues this poses. For example, Rossomanno et al [39] found that only 51% of officers reported meeting recommendations for physical activity at their study end and theorized that it was possible that participants may have lacked motivation, time, and incentive to continue the training necessary past end date. Recent UK research in this area involving 2 other UK police forces [36,40] has included a physical activity wearables study, which used a combination of a Fitbit activity monitor and *Bupa Boost* smartphone app to promote physical activity and reduce sedentary behavior in police officers.

In client meetings, another of the existing solutions listed was investing in retreats focusing on mental health and well-being. However, this particular aspect is currently limited to the numbers who can take advantage as opposed to the number of staff working for the force overall. The College of Policing recently conducted a randomized controlled trial, giving 5 other police forces access to either Headspace or Mindfit Cop. This study found that after 6 months, the average well-being, life satisfaction, resilience, and performance of individuals in the intervention groups improved compared with the control group [41]. Whereas a UK-based mobile app (Backup Buddy) allowing police officers to informally view static audio and video information and signposted support options on common mental health issues is used by 14 other UK forces [42]. No research data are currently available on its usage and impact. A research study is also currently in progress to better understand police officer experiences of the 87% well-being app, which supports employee well-being strategies [43].

In the United Kingdom, the National Police Wellbeing Service [44] covers 8 core elements to deliver tailored support and guidance for policing. Although offering a broad approach to support the health and well-being of policing personnel, it does not presently incorporate or recommend the use of digital solutions [34]. From the literature reviewed, each UK force has a certain degree of autonomy in promoting health and well-being and working with the research community and external providers. This can lead to differences in approaches and solutions used, as pointed out in the analysis of the Blue Light Wellbeing Framework [45]. Initiatives within the United Kingdom have seemingly worked in silos, with subsets of forces piloting interventions before a wider roll out—note the number of differing ongoing mental health initiatives listed earlier. This approach can reduce opportunities for collaboration and knowledge exchange and increase the chances of multiple initiatives overlapping—targeting the same areas at the same time with different technology and devices.

#### Gathering Views About How Technology Could Be Used More Effectively

The 4 highest prioritized domains were food and diet, activity, fluid intake, and sleep (Textbox 1). Behavior change is likely to play a large part in making an app focusing on health and well-being successful, with the suggestion that increased implementation of behavioral change techniques (BCTs) could improve interventions and achieve higher levels of user engagement [46]. Evidence also suggests that interventions incorporating multiple BCTs are more effective in meeting the challenge of long-term, sustainable change than those using a few or single BCT [47]. Antezana et al [46] suggested that project teams should embrace different BCTs and establish synergies by linking different features. This seems to be borne out in the limited number of BCTs found in many apps [32], suggesting an opportunity for improvement in new designs. It is also suggested to avoid the pitfalls of badly designed and unengaging interventions that project teams gather in-depth feedback about users’ views of all elements of interventions so they and developers can understand what users required [23,48]—part of the reason for our human-centered approach to this study [23].

As a 24/7 service, police officers typically work a 40-hour week, in shifts, including weekends and bank holidays [49]. Shift patterns might have 12 hours between shifts and have a minimum of either a continuous 24-hour rest period each week, or an uninterrupted rest period of 48 hours in any reference period of 14 days. They might also need to support the delivery of operational demand. A common pattern for policing is 2 day shifts, 2 late shifts and 2 night shifts, followed by 4 days off, referred to as a *six on four off*. Currently, few apps allow for customization concerning these patterns to help meet the needs and requirements of shift workers [33] and specifically police officers [1-5]. This correlates with participants’ responses and literature demonstrating a dearth of relevant research being published in this area [34], which our project helps to redress.

Mirroring the thoughts of Antezana et al [46] on multiple BCTs, participants noted that a multifunctional tool would be more beneficial to them than focusing on a particular individual element. Many current apps focus on one specific area of health and well-being, meaning that to focus on general health and well-being, the user needs to download and use different apps rather than personalizing just one. An individual could theoretically customize a suite of apps to use for managing aspects of health they were interested in or adopt a generic multifunctional tool such as Apple or Samsung Health as a very small number of participants had (Textbox 1). However, a much larger proportion of participants felt that this did not provide a suitable solution when coupled with the particular lifestyles and working environments of their jobs [1-5].

In terms of key feature requirements, data showed that participants wanted the ability to visualize how their behavior might be changing and how aspects used could be personalized, such as those relating to changes in individual shift patterns. The option of personalized customization is recognized as one of the advantages of mHealth interventions [38].

Half of the participants stated that they did not enjoy gamification within an app, and a large emphasis on gamification in association with goal-setting features would be unwise. As people get older, they are less likely to have experience of using digital games and the nuances of gamification elements (having not grown up within this culture). They might have different assumptions [50], making it essential to account for their experience with games to conceptualize a successful and suitable gamified intervention. Moreover, goals and priorities differ between younger and older people [51], which might have an impact on what motivates or affects their enjoyment of gamification. It is also possible that elements of the culture of working within the police might view gaming as a frivolous activity [37], as evidenced in some participant responses. However, it could be possible to implement less intrusive gamification elements, such as goals for the user, without a focus on teams or competition. For example, previous research looking at a number of physical activity apps noted that combinations of BCTs embedded within gamified apps were reported as self-monitoring and goal setting, with the addition of either a focus on past success or nonspecific rewards and incentives [52].

Participants also viewed the ability to enter diary-based information, such as notes associated with different shift times, into the app as being very useful. This area deserves further investigation, especially in relation to mental health.

In terms of customizable notifications, participants thought they would help them to remember to do certain tasks, whereas shift-related customized reminders were also reported as likely to be very helpful. As this particular area seems polarizing based on individual work-life practices highlighted, a good middle ground would allow users to customize the notifications received. This way, it would not be off-putting for users who either liked or disliked checking an app regularly. This would also allow users to be notified about areas of health and well-being that they designated as being of interest—a popular technique for incorporation into digital health apps [52-54].

### Limitations

A limitation of this study is that for the age descriptive calculation (Table 1), police officers usually retire at a much younger age in the United Kingdom (usually between 50 and 55 years) than the current UK age of 65 years used, which is when police staff might retire. As we did not formerly differentiate between police officers and staff during data collection, this might mean that the upper limit used for this calculation is not entirely representative. Although the authors believe that the results of this study can be generalized for other police forces across the country, we acknowledge that there is a limitation of only accessing data from a subsection of one regional UK police force, which might have inherent organizational biases toward health and well-being. However, this approach can be expanded upon to cover more regions in due course. Concerning bias with purposeful sampling—where the belief is that qualitative research should describe the medium or the norm—the point to underline is that new phenomena are being described, so we needed to purposively select the best examples of what we were interested in. This gave us the clearest cases with the least *noise* or extraneous errors and allowed for the identification of characteristics and boundaries [55]. A further limitation of this study is the greater number of female responses (134/213, 62.9%) than male responses when compared with the police workforce in England and Wales numbers as of 2016, which noted that only 28.6% of all officers were female [56]. We will attempt to improve upon this gender imbalance in future stages of the study.

### Conclusions

For police officers and staff, research data suggest that there is a previously unidentified requirement for a mobile app that provides an easily accessible platform for them to use regardless of the current location. One that could provide guidelines on diet, lifestyle habits, and health behavior to help the user make informed decisions to assist in personalized behavior change. A multifunctional app, which aligns with the irregular shift patterns of its users, can be customized effectively for their specific needs. More detailed requirements gathering is therefore expected to take place while funding is sought for the creation and prototyping of a solution. This work will include drafting initial requirements and preferences for devising a new targeted solution in this area as a starting point for future prototyping, then creating and prototyping a solution. It will continue to ensure the participation of police officers and staff, working toward a human-centered design methodology at each stage of the development cycle.

## Abbreviations

BCT: behavioral change technique
CPTSD: complex posttraumatic stress disorder
mHealth: mobile health
PTSD: posttraumatic stress disorder

## References

[1] Violanti JM, Charles LE, McCanlies E, Hartley TA, Baughman P, Andrew ME, Fekedulegn D, Ma CC, Mnatsakanova A, Burchfiel CM (2017). Police stressors and health: a state-of-the-art review. Int J Police Strat Manag.

[2] Demou E, Hale H, Hunt K (2020). Understanding the mental health and wellbeing needs of police officers and staff in Scotland. Police Pract Res.

[3] Ma CC, Andrew ME, Fekedulegn D, Gu JK, Hartley TA, Charles LE, Violanti JM, Burchfiel CM (2015). Shift work and occupational stress in police officers. Saf Health Work.

[4] Galbraith N, Boyda D, McFeeters D, Galbraith V (2021). Patterns of occupational stress in police contact and dispatch personnel: implications for physical and psychological health. Int Arch Occup Environ Health.

[5] Kula S (2016). Occupational stress, supervisor support, job satisfaction, and work-related burnout: perceptions of Turkish National Police (TNP) members. Police Pract Res.

[6] Mayhew C (2001). Occupational health and safety risks faced by police officers. Trends & Issues in Crime and Criminal Justice.

[7] Fox J, Desai MM, Britten K, Lucas G, Luneau R, Rosenthal MS (2012). Mental-health conditions, barriers to care, and productivity loss among officers in an urban police department. Conn Med.

[8] Miller J (2018). Police Care UK.

[9] Brewin CR, Miller JK, Soffia M, Peart A, Burchell B (2020). Posttraumatic stress disorder and complex posttraumatic stress disorder in UK police officers. Psychol Med.

[10] Zimmerman F (2012). Cardiovascular disease and risk factors in law enforcement personnel: a comprehensive review. Cardiol Rev.

[11] Wirth M, Vena J, Burch J, Violanti JM (2014). Risk of cancer incidence and cancer mortality among police officers. Dying for the Job: Police Work Exposure and Health.

[12] Ramey S (2002). Relationship between cardiovascular disease morbidity, risk factors, and stress in a law enforcement cohort. Retrospective Thesis and Dissertations, Iowa State University.

[13] Ramey SL (2003). Cardiovascular disease risk factors and the perception of general health among male law enforcement officers: encouraging behavioral change. AAOHN J.

[14] Burton AK, Tillotson KM, Symonds TL, Burke C, Mathewson T (1996). Occupational risk factors for the first-onset and subsequent course of low back trouble. A study of serving police officers. Spine (Phila Pa 1976).

[15] United Kingdom. Institute for Health Metrics and Evaluation.

[16] Roger VL, Go AS, Lloyd-Jones DM, Adams RJ, Berry JD, Brown TM, Carnethon MR, Dai S, de Simone G, Ford ES, Fox CS, Fullerton HJ, Gillespie C, Greenlund KJ, Hailpern SM, Heit JA, Ho PM, Howard VJ, Kissela BM, Kittner SJ, Lackland DT, Lichtman JH, Lisabeth LD, Makuc DM, Marcus GM, Marelli A, Matchar DB, McDermott MM, Meigs JB, Moy CS, Mozaffarian D, Mussolino ME, Nichol G, Paynter NP, Rosamond WD, Sorlie PD, Stafford RS, Turan TN, Turner MB, Wong ND, Wylie-Rosett J, American Heart Association Statistics CommitteeStroke Statistics Subcommittee (2011). Heart disease and stroke statistics--2011 update: a report from the American Heart Association. Circulation.

[17] Velavan TP, Meyer CG (2020). The COVID-19 epidemic. Trop Med Int Health.

[18] Baker C, Brown JS Research Briefing - Coronavirus: a history of English lockdown laws. House of Commons Library.

[19] COVID-19 restrictions. College of Policing.

[20] Stogner J, Miller BL, McLean K (2020). Police stress, mental health, and resiliency during the COVID-19 pandemic. Am J Crim Justice.

[21] Frenkel MO, Giessing L, Egger-Lampl S, Hutter V, Oudejans RR, Kleygrewe L, Jaspaert E, Plessner H (2021). The impact of the COVID-19 pandemic on European police officers: Stress, demands, and coping resources. J Crim Justice.

[22] De Camargo C (2021). ‘It’s tough shit, basically, that you're all gonna get it': UK virus testing and police officer anxieties of contracting COVID-19. Policing Soc.

[23] Yardley L, Morrison L, Bradbury K, Muller I (2015). The person-based approach to intervention development: application to digital health-related behavior change interventions. J Med Internet Res.

[24] Chung KC, Pillsbury MS, Walters MR, Hayward RA (1998). Reliability and validity testing of the Michigan Hand Outcomes Questionnaire. J Hand Surg Am.

[25] Fugard AJ, Potts HW (2015). Supporting thinking on sample sizes for thematic analyses: a quantitative tool. Int J Soc Res Methodol.

[26] Caelli K, Ray L, Mill J (2016). ‘Clear as Mud’: Toward greater clarity in generic qualitative research. Int J Qual Methods.

[27] Braun V, Clarke V (2006). Using thematic analysis in psychology. Qual Res Psychol.

[28] The Global Challenge. Virgin Pulse.

[29] Sullivan AN, Lachman ME (2016). Behavior change with fitness technology in sedentary adults: a review of the evidence for increasing physical activity. Front Public Health.

[30] Jimenez P, Bregenzer A (2018). Integration of eHealth tools in the process of workplace health promotion: proposal for design and implementation. J Med Internet Res.

[31] Chuah SH, Rauschnabel PA, Krey N, Nguyen B, Ramayah T, Lade S (2016). Wearable technologies: The role of usefulness and visibility in smartwatch adoption. Comput Hum Behav.

[32] McKay FH, Wright A, Shill J, Stephens H, Uccellini M (2019). Using health and well-being apps for behavior change: a systematic search and rating of apps. JMIR Mhealth Uhealth.

[33] Nunes F, Ribeiro J, Braga C, Lopes P (2018). Supporting the self-care practices of shift workers. Proceedings of the 17th International Conference on Mobile and Ubiquitous Multimedia.

[34] Marston HR, Hadley R, Pike G, Hesketh I (2020). Police J.

[35] Cole JA, Tully MA, Cupples ME (2015). "They should stay at their desk until the work's done": a qualitative study examining perceptions of sedentary behaviour in a desk-based occupational setting. BMC Res Notes.

[36] Buckingham SA, Morrissey K, Williams AJ, Price L, Harrison J (2020). The Physical Activity Wearables in the Police Force (PAW-Force) study: acceptability and impact. BMC Public Health.

[37] Velazquez E, Hernandez M (2019). Effects of police officer exposure to traumatic experiences and recognizing the stigma associated with police officer mental health. Policing Int J.

[38] Huang Y, Benford S, Blake H (2019). Digital interventions to reduce sedentary behaviors of office workers: scoping review. J Med Internet Res.

[39] Rossomanno C, Herrick J, Kirk S (2012). A 6-month supervised employer-based minimal exercise program for police officers improves fitness. J Streng Condit Res.

[40] Buckingham S, Morrissey K, Williams A, Price L, Harrison J (2019). OP67 The physical activity wearables in the police force (PAW-force) trial: feasibility, acceptability and impact. J Epidemiol Community Health.

[41] Fitzhugh H (2020). Does mindfulness work in policing?. GOV.UK Blog: What Works.

[42] (2020). Blog: Backup Buddy: Police mental health app. National Police Wellbeing Service.

[43] 87% app pilot study participant information. Police Federation.

[44] National Police Wellbeing Service Introduction. National Police Wellbeing Service.

[45] (2018). Wellbeing in policing: Blue light wellbeing framework. University of Central Lancashire and Lancashire Constabulary.

[46] Antezana G, Venning A, Blake V, Smith D, Winsall M, Orlowski S, Bidargaddi N (2020). An evaluation of behaviour change techniques in health and lifestyle mobile applications. Health Informatics J.

[47] Pagoto S, Bennett GG (2013). How behavioral science can advance digital health. Transl Behav Med.

[48] Yardley L, Morrison L, Muller I, Bradbury K, Hagger M, Cameron K, Hamilton L, Hankonen N, Lintunen T (2020). Maximizing user engagement with behavior change interventions. The Handbook of Behavior Change (Cambridge Handbooks in Psychology).

[49] Force Policy - Shift working and working time for police staff. West Yorkshire Police.

[50] Brox E, Konstantinidis ST, Evertsen G (2017). User-centered design of serious games for older adults following 3 years of experience with exergames for seniors: a study design. JMIR Serious Games.

[51] Altmeyer M, Lessel P, Krüger A (2018). Investigating gamification for seniors aged 75+. Proceedings of the 2018 Designing Interactive Systems Conference.

[52] Pickering K, Pringle A (2018). Gamification for physical activity behaviour change. Perspect Public Health.

[53] Thomas S, Pulman A, Dogan H, Jiang N, Thomas P, Passmore D, Pretty K, Davies Smith A, Fairbanks A Creating a digital toolkit to reduce fatigue and promote quality of life in multiple sclerosis: mixed methods. JMIR Preprints..

[54] Attwood S, Parke H, Larsen J, Morton KL (2017). BMC Public Health.

[55] Morse J (2012). Qualitative Health Research: Creating a New Discipline.

[56] (2016). Police Workforce, England and Wales, 31 March 2016. Home Office Statistical Bulletin 05/16.

